# Supplementary material for: Identification of distinct loci for de novo DNA methylation by DNMT3A and DNMT3B during mammalian development
Source: Nat Commun. 2020 Jun 24;11:3199. doi: 10.1038/s41467-020-16989-w (PMC7314859; doi:10.1038/s41467-020-16989-w)
Supplement: Supplementary file 1 — Supplementary Information [file 41467_2020_16989_MOESM1_ESM.pdf]

Identification of distinct loci for *de novo* DNA methylation by DNMT3A and DNMT3B during mammalian development

Yagi et al.

Inventory of Supplementary Information

Supplementary Figures (Supplementary Figures 1-9)

Legends to Supplementary Figures

Supplementary Figure 1

a

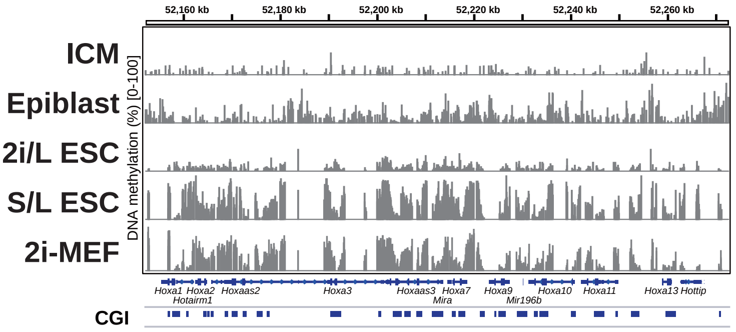

b

Cell Type	Sample Name	Gender	Passage No.	Genetic Background	Method	Seq Type	Read No. (Mb)	C-T conversion rate (%)
MEF	2i-MEFs WT rep1	XX	p1	129/MSM (♀/♂)	MethylC-seq	Hiseq Pair	81.3 (×2)	99.08
MEF	2i-MEFs 3a KO rep1	XX	p1	129/MSM (♀/♂)	MethylC-seq	Hiseq Pair	97.8 (×2)	99.02
MEF	2i-MEFs 3b KO rep1	XX	p1	129/MSM (♀/♂)	MethylC-seq	Hiseq Pair	90.5 (×2)	99.06
MEF	2i-MEFs WT rep1	XX	p1	129/MSM (♀/♂)	WGBS	Hiseq Pair	77.7 (×2)	99.52
MEF	2i-MEFs 3a KO rep1	XX	p1	129/MSM (♀/♂)	WGBS	Hiseq Pair	105.4 (×2)	99.39
MEF	2i-MEFs 3b KO rep1	XX	p1	129/MSM (♀/♂)	WGBS	Hiseq Pair	93.8 (×2)	99.44
MEF	2i-MEFs WT rep2	XX	p1	129/MSM (♀/♂)	MethylC-seq	Hiseq Pair	36.6 (×2)	98.99
MEF	2i-MEFs 3a KO rep2	XX	p1	129/MSM (♀/♂)	MethylC-seq	Hiseq Pair	37.4 (×2)	99.01
MEF	2i-MEFs 3b KO rep2	XX	p1	129/MSM (♀/♂)	MethylC-seq	Hiseq Pair	31.8 (×2)	99
MEF	2i-MEFs WT rep2	XX	p1	129/MSM (♀/♂)	WGBS	Hiseq Pair	121.9 (×2)	99.51
MEF	2i-MEFs 3a KO rep2	XX	p1	129/MSM (♀/♂)	WGBS	Hiseq Pair	136.4 (×2)	99.53
MEF	2i-MEFs 3b KO rep2	XX	p1	129/MSM (♀/♂)	WGBS	Hiseq Pair	122.6 (×2)	99.55
Brain	Brain 3a WT	XY	-	B6	MethylC-seq	Hiseq Pair	27.1 (×2)	99.46
Brain	Brain 3a KO	XY	-	B6	MethylC-seq	Hiseq Pair	30.2 (×2)	99.45
Limb	Limb 3a WT	XY	-	B6	MethylC-seq	Hiseq Pair	26.5 (×2)	99.44
Limb	Limb 3a KO	XY	-	B6	MethylC-seq	Hiseq Pair	25.9 (×2)	99.45
Heart	Heart 3a WT	XY	-	B6	MethylC-seq	Hiseq Pair	40.8 (×2)	99.42
Heart	Heart 3a KO	XY	-	B6	MethylC-seq	Hiseq Pair	23.5 (×2)	99.44
Lung	Lung 3a WT	XY	-	B6	MethylC-seq	Hiseq Pair	29.3 (×2)	99.44
Lung	Lung 3a KO	XY	-	B6	MethylC-seq	Hiseq Pair	26.1 (×2)	99.44
Liver	Liver 3a WT	XY	-	B6	MethylC-seq	Hiseq Pair	33.2 (×2)	99.45
Liver	Liver 3a KO	XY	-	B6	MethylC-seq	Hiseq Pair	23.3 (×2)	99.47

c

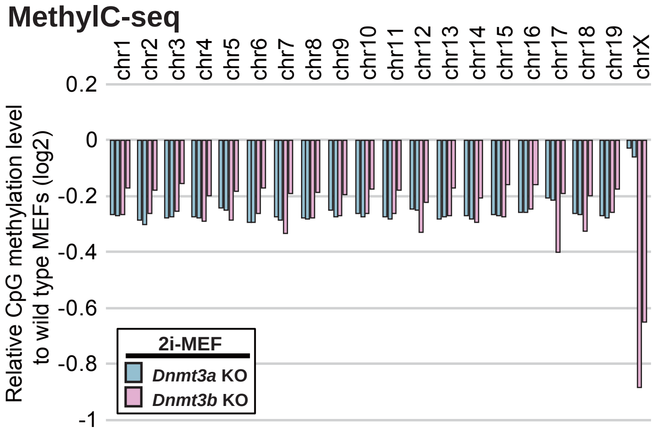

d

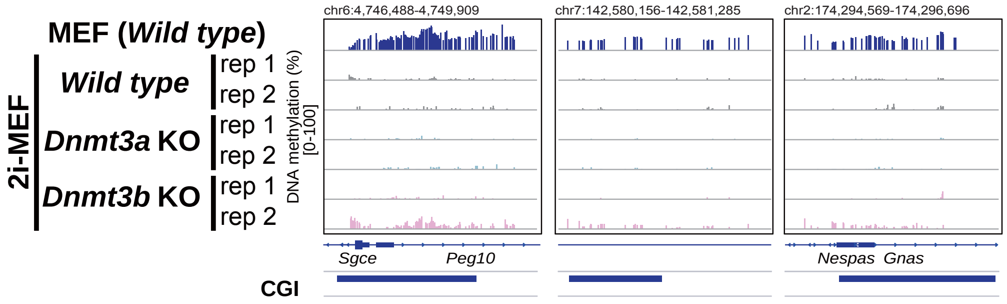

Supplementary Figure 1:
2i/L ES cells for dissecting unique targets of *de novo* methylation by DNMT3 enzymes

a: CpG methylation levels at the *Hoxa* cluster. Each bar indicates a CpG site, and bar height represents methylation percentage (0–100%). Locations of genes and CGIs are indicated below. WGBS data of ICM and epiblast were obtained from GSE84236. MethylC-seq data were used for 2i/L ES cells, S/L ES cells and 2i-MEFs.
b: Total numbers of WGBS and MethylC-seq sequenced reads used in these analyses.
c: Relative CpG methylation level ($\log_2$) at each chromosome in *Dnmt3* KO 2i-MEFs vs. wild-type 2i-MEFs, as determined by MethylC-seq. Data from two independent experiments are shown. Note that the level at the X chromosome is markedly lower in *Dnmt3b* KO MEFs.
d: CpG methylation levels at imprinting control regions. Each bar indicates a CpG site, and bar height represents methylation percentage (0–100%). Locations of genes are indicated below. MethylC-seq data for wild type MEF was obtained from GSE84165.

Supplementary Figure 2

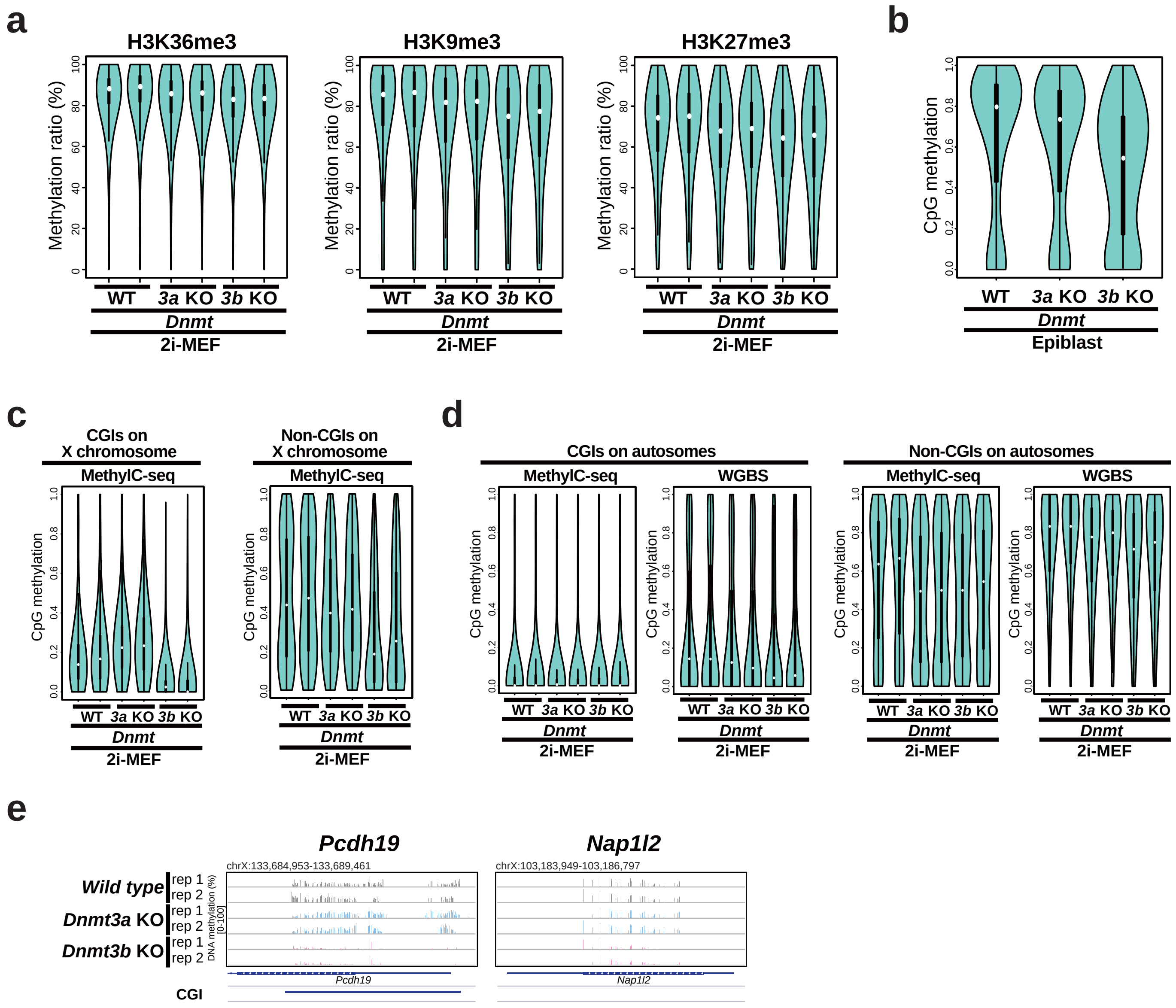

Supplementary Figure 2:
Predominant role of DNMT3B in global *de novo* DNA methylation on the X chromosome

a: CpG methylation levels at H3K36me3, H3K9me3, and H3K27me3 marked sites in WT, *Dnmt3a* KO, and *Dnmt3b* KO 2i-MEFs, from two independent WGBS experiments. White dots indicate median methylation levels. Black bars and the lines stretched from the bar represent interquartile range (IQR) and the lower/upper adjacent values (±1.5 IQR), respectively. MEF ChIP-seq data for H3K36me3, H3K27me3, and H3K9me3 were obtained from GSE90895.
b: CpG methylation levels at loci that were differentially methylated between ICM and epiblast (Fig. 1b, see the methods section for details) in WT, *Dnmt3a* KO, and *Dnmt3b* KO epiblasts. RRBS data for epiblasts were obtained from GSE84236.
c: CpG methylation levels at CGIs and non-CGIs on the X chromosome in WT, *Dnmt3a* KO, and *Dnmt3b* KO 2i-MEFs, as determined by MethylC-seq. Data from two independent experiments are shown. White dots indicate median methylation levels. DNA methylation levels at both CGIs and non-CGIs on the X chromosome were substantially reduced in *Dnmt3b* KO MEFs.
d: CpG methylation levels at CGIs and non-CGIs on autosomal chromosomes in WT, *Dnmt3a* KO, and *Dnmt3b* KO 2i-MEFs, as determined by MethylC-seq and WGBS. Data from two independent experiments are shown.
e: Representative regions on the X chromosome, which exhibit reduced DNA methylation levels in *Dnmt3b* KO 2i-MEFs. Data from two independent experiments are shown. Each bar indicates a CpG site, and bar height represents methylation percentage (0–100%). Locations of genes and CGIs are indicated below. *Dnmt3b* KO 2i-MEFs exhibited hypomethylation at both CGIs and non-CGIs.

Supplementary Figure 3

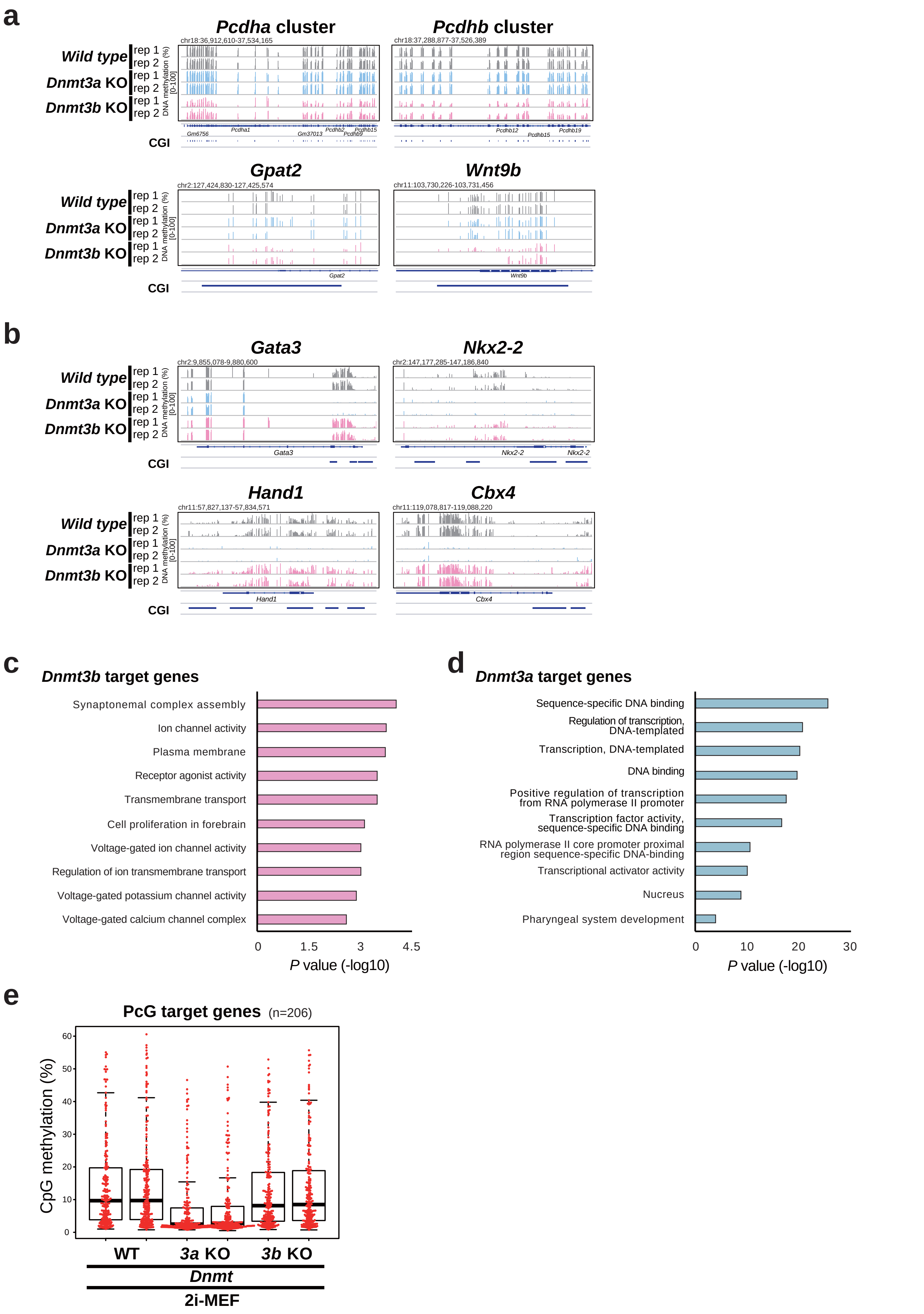

Supplementary Figure 3:
Target genes of *de novo* methylation by DNMT3A and DNMT3B

a: DNMT3B target sites. Data from two independent experiments are shown. Each bar indicates a CpG site, and bar height represents methylation percentage (0–100%). Locations of genes and CGIs are indicated below.
b: DNMT3A target sites. Developmental genes exhibited reduced methylation levels in *Dnmt3a* KO 2i-MEFs.
c: GO term enrichments in the DNMT3B-specific target genes listed in Supplementary Table 5. The top 10 terms are listed. GO analysis was performed using DAVID Bioinformatics Resource 6.8. Analysis categories were narrowed down to GOTERM_BP_DIRECT, GOTERM_CC_DIRECT, and GOTERM_MF_DIRECT.
d: GO term enrichments in DNMT3A-specific target genes listed in Supplementary Table 3. The top 10 terms are listed. GO analysis was performed using DAVID Bioinformatics Resource 6.8. Analysis categories was narrowed down to GOTERM_BP_DIRECT, GOTERM_CC_DIRECT, and GOTERM_MF_DIRECT. Note that several terms related to development, DNA binding, and transcription were enriched.
e: Box plots of CpG methylation levels at PcG target developmental genes in WT, *Dnmt3a* KO, and *Dnmt3b* KO 2i-MEFs, as determined by MethylC-seq. Data from two independent experiments are shown. The bold black lines indicate median methylation levels. Bottom and top of the box are lower and upper quartiles, respectively. Whiskers extend to ±1.5 IQR. Statistical analysis was performed by two-sided Wilcoxon signed-rank test, *3a* KO vs WT (mean methylation levels of each PcG target gene): $p = 1.0\times10^{-47}$, *3b* KO vs WT (mean methylation levels of each PcG target gene): $p = 6.6\times10^{-16}$.

Supplementary Figure 4

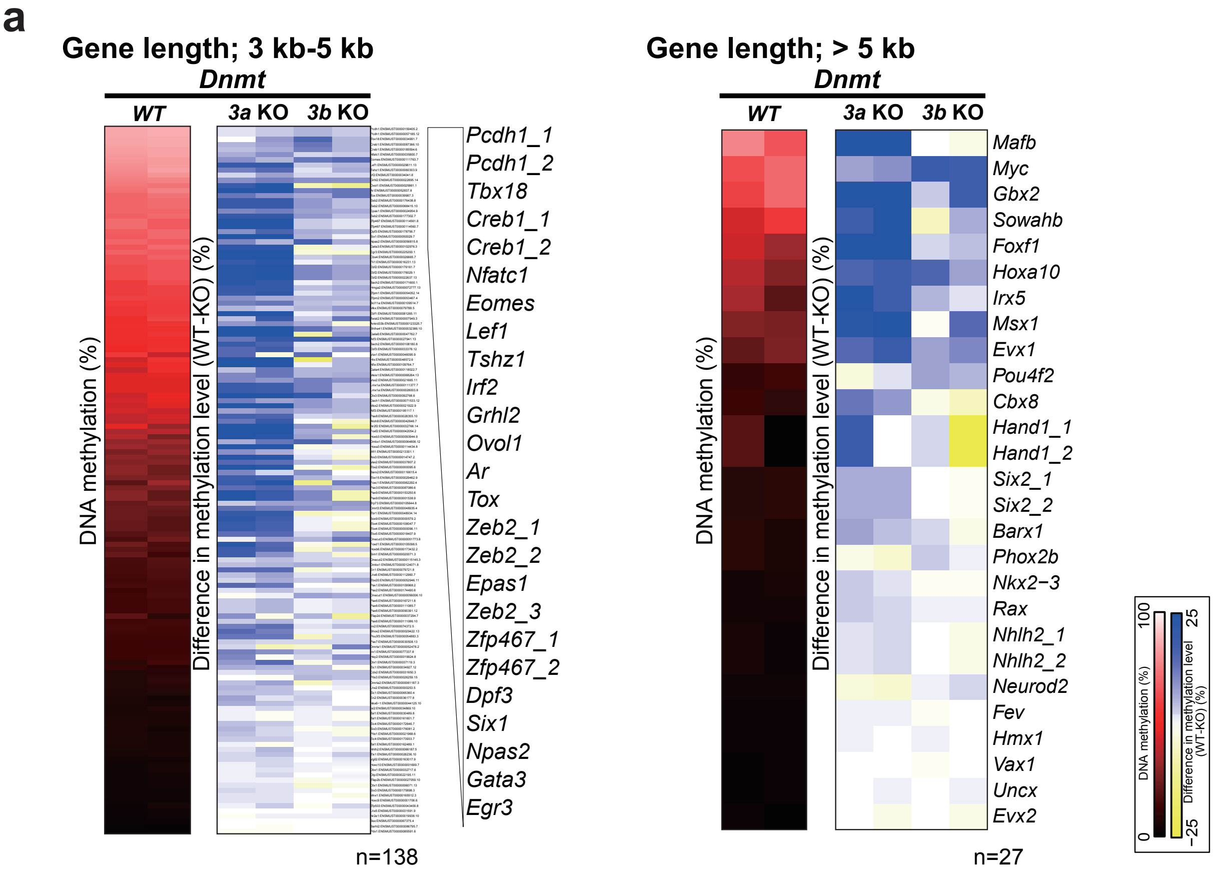

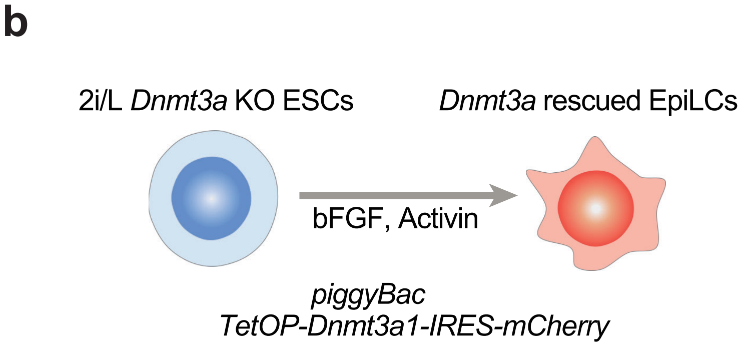

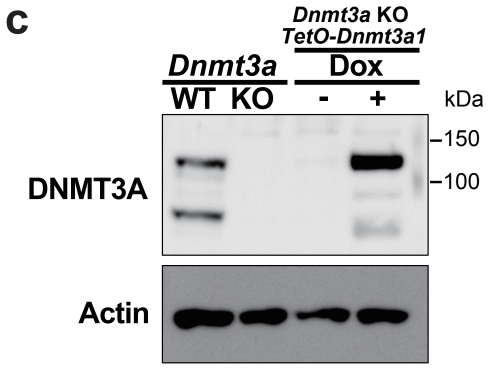

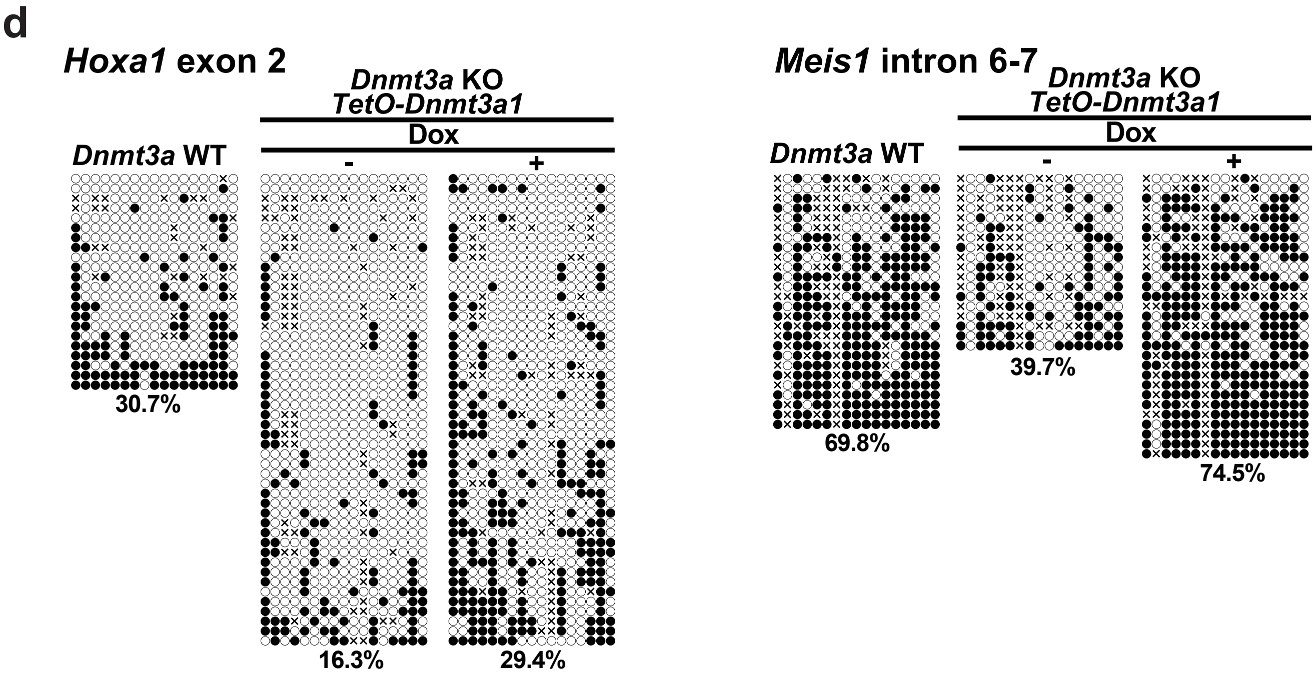

Supplementary Figure 4:

Ectopic expression of *Dnmt3a1* rescues the reduction in DNA methylation levels at PcG target genes in *Dnmt3a* KO cells

a: CpG methylation levels at PcG target developmental genes with different gene lengths in WT 2i-MEFs, as determined by two independent MethylC-seq experiments. Mean methylation levels at CpG sites within regions (from TSS to TES) and the methylation difference between WT and each *Dnmt3* KO 2i-MEFs are shown [Shorter genes: *3a* KO vs WT : $p = 2.1\times10^{-5}$, *3b* KO: $p = 2.3\times10^{-1}$ (two-sided Wilcoxon signed-rank test), Longer genes: *3a* KO: $p = 1.0\times10^{-36}$, *3b* KO: $p = 3.9\times10^{-16}$ (two-sided Wilcoxon signed-rank test)]. Genes are sorted by the methylation levels in the WT. Color scales indicate CpG methylation levels and their differences, respectively.

b: Strategy for rescue experiments in *Dnmt3a* KO 2i/L cells. A doxycycline (Dox)-inducible *piggyBac* vector containing *tetOP-Dnmt3a1-IRES-mCherry-EF1-rtTA-IRES-Neo* (*PB-Dnmt3a*) was used for induction of *Dnmt3a1*.

c: Western blot analysis for DNMT3A. The original 2i/L ES cells were used as the WT sample, and *Dnmt3a* KO 2i/L ES cells as the KO sample. Ectopic expression of DNMT3A1 was induced in *Dnmt3a* KO 2i/L ES cells following Dox exposure (rescue sample). Each ES cell line was differentiated into EpiLCs for 3 days prior to western blot analysis. Uncut gel images are available in a Source Data file.

d: Conventional bisulfite sequencing of *Hoxa1* and *Meis1* in EpiLCs. Open circles represent unmethylated CpGs, closed circles represent methylated CpGs, and crosses indicate undetermined methylation status. Dox+-rescued cells exhibited methylation levels similar to those of WT cells.

Supplementary Figure 5

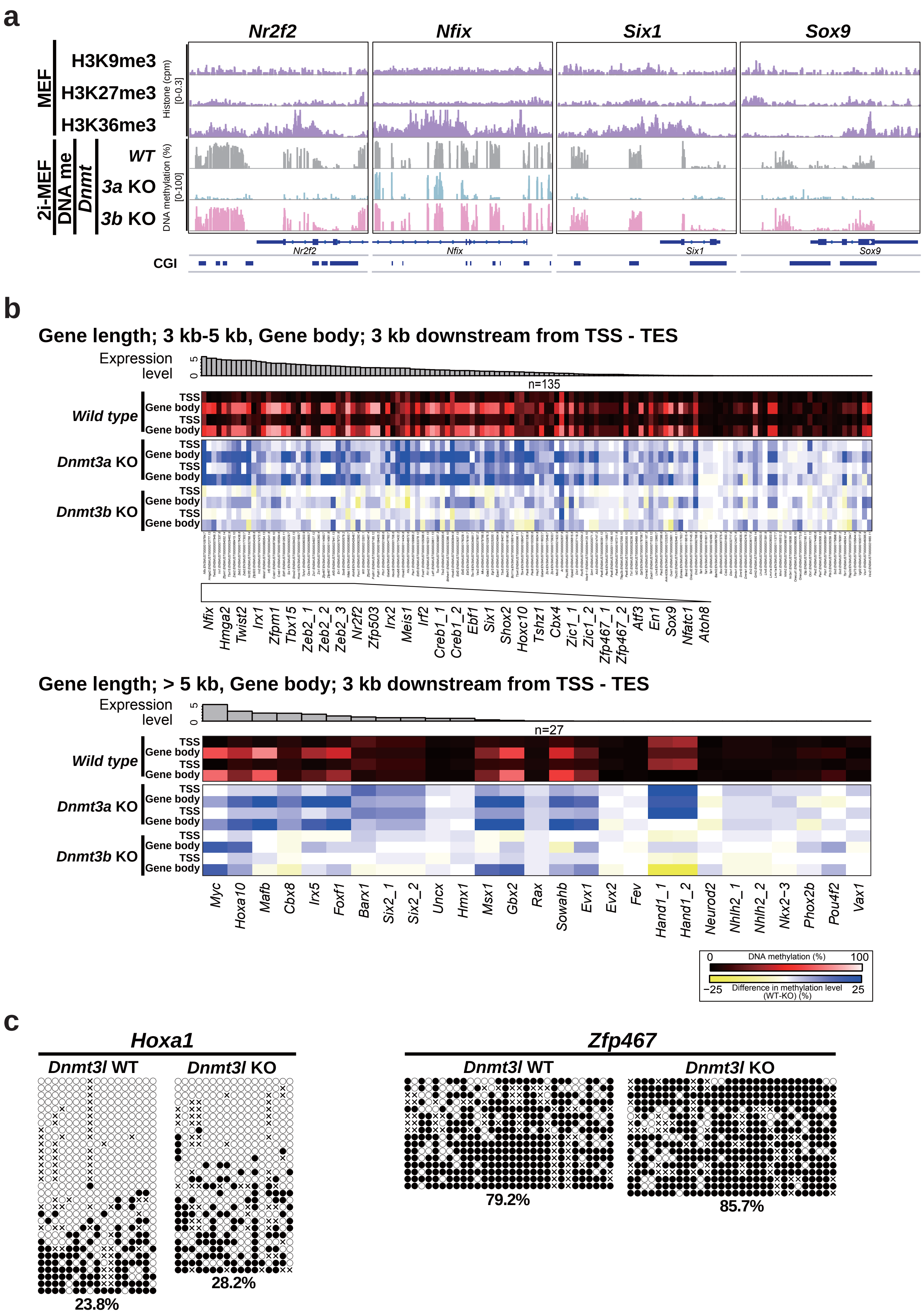

Supplementary Figure 5:
DNMT3A-mediated *de novo* methylation at PcG target genes in MEFs

a: Histone modifications at DNMT3A target genes in MEFs. H3K36me3-marked PcG target genes exhibit reduced DNA methylation levels in *Dnmt3a* KO 2i-MEFs. Locations of genes and CGIs are indicated below. ChIP-seq data of MEFs for H3K36me3, H3K27me3, and H3K9me3 were from GSE90895.

b: CpG methylation levels within TSS ± 1000 bp and gene body (3 kb downstream from TSS to TES) of PcG target genes with different gene lengths, as determined by two independent MethylC-seq experiments. Mean methylation levels at CpG sites within the regions (upper panel) and the difference in methylation between WT and each type of *Dnmt3* KO 2i-MEFs (lower panel) are shown [Shorter genes: two-sided Wilcoxon signed-rank test, *3a* KO vs WT (TSS): $p = 2.1\times10^{-5}$, *3b* KO vs WT (TSS): $p = 2.3\times10^{-1}$, *3a* KO vs WT (Gene body): $p = 7.5\times10^{-8}$, *3b* KO vs WT (Gene body): $p = 1.9\times10^{-1}$. Longer genes: two-sided Wilcoxon signed-rank test, *3a* KO vs WT (TSS): $p < 2.2\times10^{-16}$, *3b* KO vs WT (TSS): $p = 1.8\times10^{-13}$, *3a* KO vs WT (Gene body): $p < 2.2\times10^{-16}$, *3b* KO vs WT (Gene body): $p = 7.4\times10^{-4}$]. Color scale indicates CpG methylation levels and their difference. PcG genes are ordered based on expression levels (FPKM value) in WT 2i-MEFs (top panel).

c: Conventional bisulfite sequencing of *Hoxa1* and *Zfp467* in *Dnmt3l* WT and KO MEFs obtained by mating *Dnmt3l* heterozygous KO mice. Open circles represent unmethylated CpGs, closed circles represent methylated CpGs, and crosses indicate undetermined methylation status. DNA methylation levels were comparable between *Dnmt3l* WT and KO MEFs.

Supplementary Figure 6

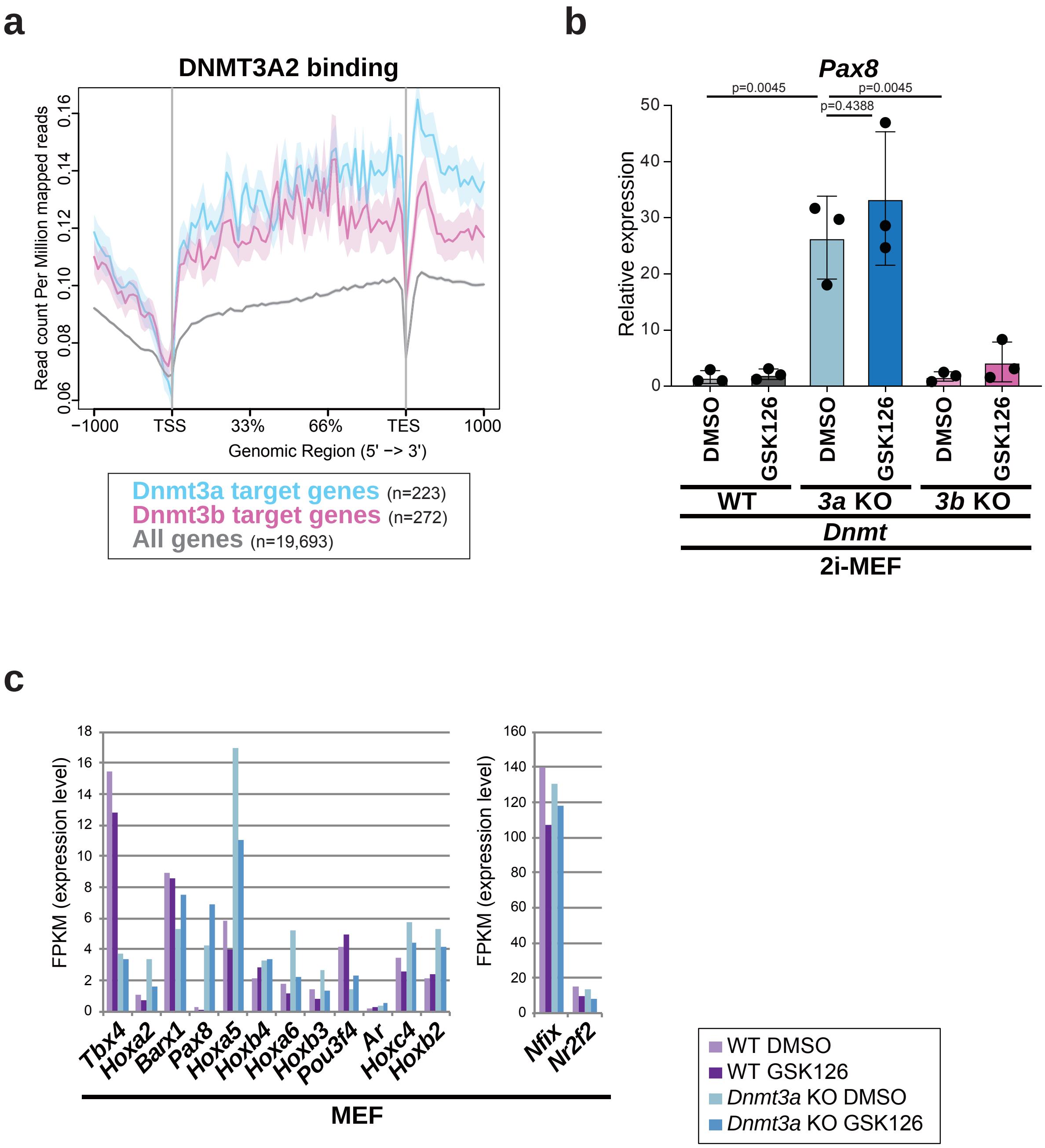

Supplementary Figure 6:
DNMT3A-mediated epigenetic repression of PcG target genes in MEFs

a: DNMT3A2 binding to target genes of *de novo* methylation by DNMT3A and DNMT3B. ChIP-seq data were obtained from GSE57413. Shading represents standard error of the mean (SEM).
b: qRT-PCR analysis for *Pax8* in 2i-MEFs with or without GSK126, EZH2 methyltransferase inhibitor treatment. *Pax8* was derepressed in *Dnmt3a* KO 2i-MEFs. No significant derepression was observed following GSK126 treatment. Data are presented as means ± SD. Mean expression levels of WT 2i-MEFs were set to 1. Statistical analysis was performed by Student's t-test (WT DMSO vs 3a KO DMSO, p=0.0045: 3a KO DMSO vs 3b KO DMSO, p=0.0045: 3a KO DMSO vs 3a KO GSK126, p=0.4388; two-sided, n = 3 biologically independent samples).
c: Expression levels (FPKM) of PcG target genes in MEFs derived from *Dnmt3a* wild-type or *Dnmt3a* KO embryos by RNA-seq. MEFs were treated with or without GSK126. Note that *Pax8* is derepressed in *Dnmt3a* KO MEFs.

Supplementary Figure 7

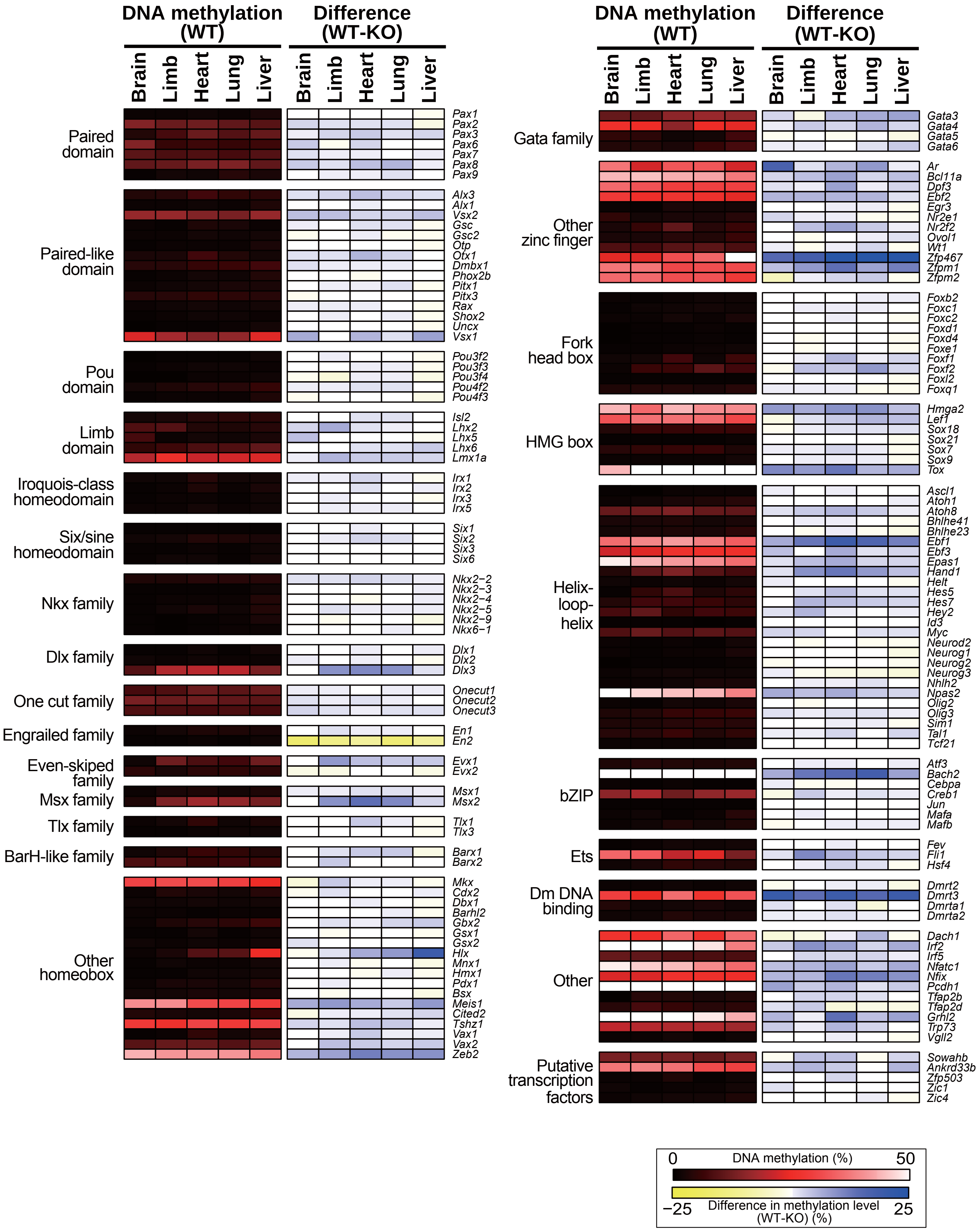

Supplementary Figure 7:

Tissue-specific reduction in DNA methylation in various PcG target genes in *Dnmt3a* KO tissues

The left panels show heatmap of mean methylation levels at CpG sites within gene bodies of various PcG target genes in different organs. The right panels show methylation differences between *Dnmt3a* WT and KO organs. Color scales indicate CpG methylation levels and their differences, respectively.

Supplementary Figure 8

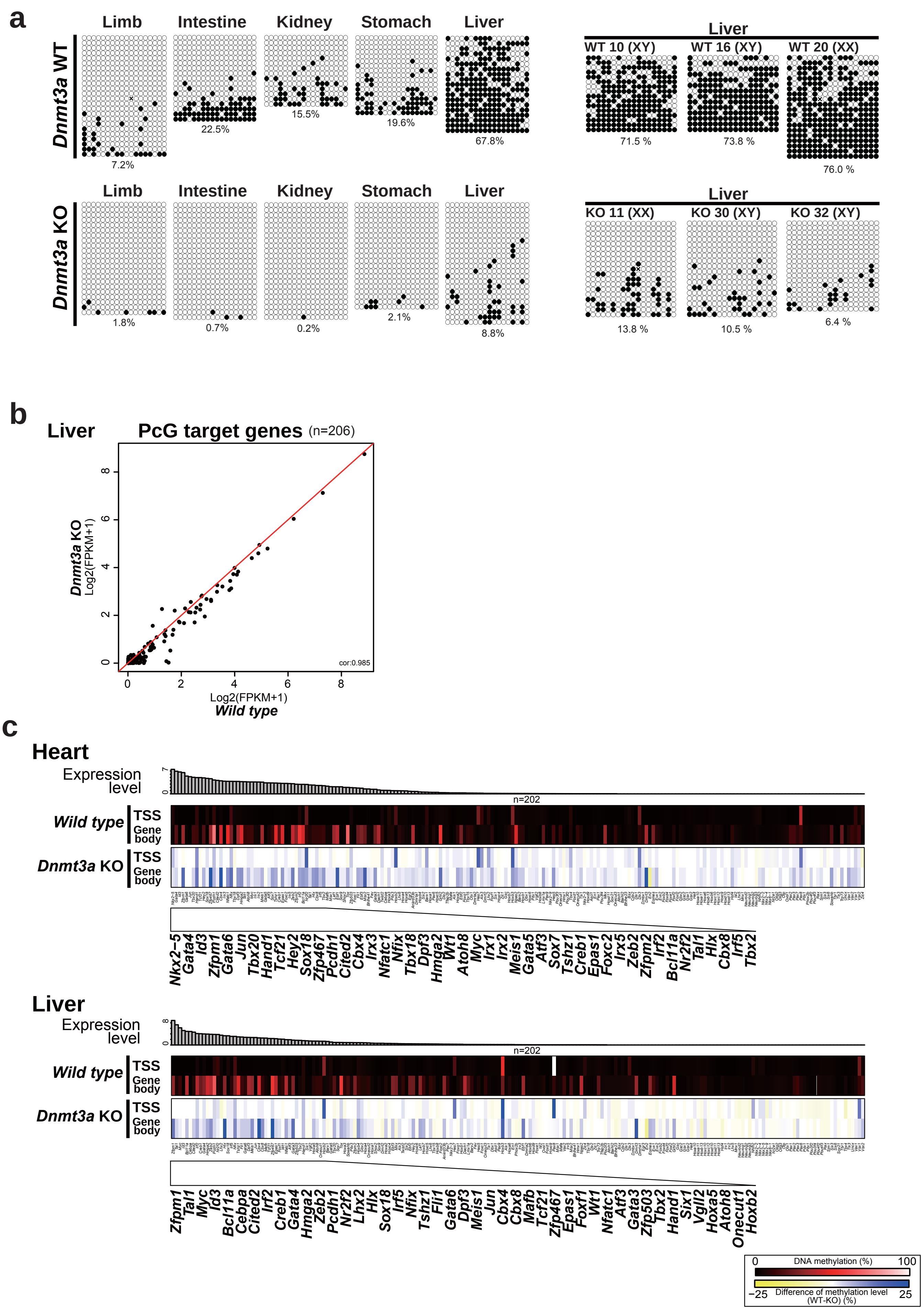

Supplementary Figure 8:

Tissue-specific regulation of DNMT3A-mediated *de novo* DNA methylation at PcG target genes

a: Conventional bisulfite sequencing of *Meis1* in *Dnmt3a* KO tissues. Tissue-specific *Meis1* methylation is reduced in *Dnmt3a* KO mice.

b: Expression levels [log2 (FPKM+1)] of PcG target genes in the liver of *Dnmt3a* WT and KO embryos, as determined by RNA-seq.

c: The upper panels show mean methylation levels at CpG sites within TSS ± 1000 bp and gene bodies (TSS-TES) of PcG target genes in *Dnmt3a* WT heart and liver. The lower panels show methylation differences between *Dnmt3a* WT and KO indicated organs. Color scales indicate CpG methylation levels and their differences. PcG genes are ordered based on their expression level (FPKM) in each organ (top panel).

Supplementary Figure 9

a

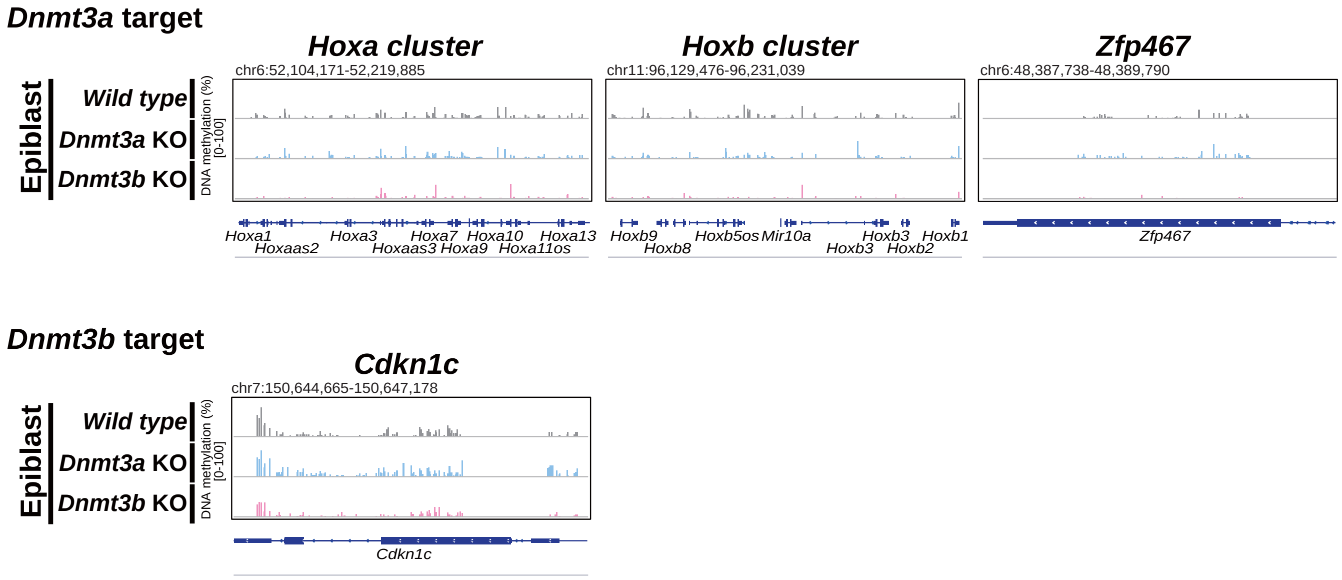

b

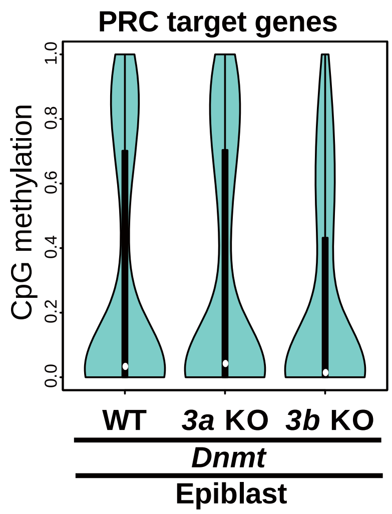

Supplementary Figure 9:
DNA hypomethylation at PcG target genes in E6.5 epiblast *in vivo*

a: CpG methylation status at DNMT3 target genes in WT, *Dnmt3a* KO, and *Dnmt3b* KO epiblasts. Each bar indicates a CG site, and bar height represents methylation percentage, as determined by RRBS (0–100%). Locations of genes are indicated below.
b: CpG methylation levels at PcG target developmental genes in WT, *Dnmt3a* KO, and *Dnmt3b* KO epiblasts. White dots indicate median methylation levels. Black bars and the lines stretched from the bar represent IQR and the lower/upper adjacent values (±1.5 IQR), respectively. RRBS data for epiblasts were obtained from GSE84236.
