# Supplementary material for: Identification of distinct loci for de novo DNA methylation by DNMT3A and DNMT3B during mammalian development
Source: Nat Commun. 2020 Jun 24;11:3199. doi: 10.1038/s41467-020-16989-w (PMC7314859; doi:10.1038/s41467-020-16989-w)
Supplement: Supplementary file 3 — Description of Additional Supplementary Files [file 41467_2020_16989_MOESM3_ESM.pdf]

Description of Additional Supplementary Files

File Name: Supplementary Data 1
Description: Identification of differentially methylated tiles (DMTs) among wild type, Dnmt3a KO and Dnmt3b KO 2i-MEFs.

File Name: Supplementary Data 2
Description: Identification of regions specifically methylated by DNMT3A.

File Name: Supplementary Data 3
Description: Identification of genes specifically methylated by DNMT3A.

File Name: Supplementary Data 4
Description: Identification of regions specifically methylated by DNMT3B.

File Name: Supplementary Data 5
Description: Identification of genes specifically methylated by DNMT3B.
