# Supplementary material for: Identification of distinct loci for de novo DNA methylation by DNMT3A and DNMT3B during mammalian development
Source: Nat Commun. 2020 Jun 24;11:3199. doi: 10.1038/s41467-020-16989-w (PMC7314859; doi:10.1038/s41467-020-16989-w)
Supplement: Supplementary file 9 — Reporting Summary [file 41467_2020_16989_MOESM9_ESM.pdf]

nature research

Corresponding author(s): Yasuhiro Yamada, Takuya Yamamoto

Last updated by author(s): May 14, 2020

Reporting Summary

Nature Research wishes to improve the reproducibility of the work that we publish. This form provides structure for consistency and transparency in reporting. For further information on Nature Research policies, see Authors & Referees and the Editorial Policy Checklist .

Statistics

For all statistical analyses, confirm that the following items are present in the figure legend, table legend, main text, or Methods section.

n/a	Confirmed	
☐	☒	The exact sample size (*n*) for each experimental group/condition, given as a discrete number and unit of measurement
☐	☒	A statement on whether measurements were taken from distinct samples or whether the same sample was measured repeatedly
☐	☒	The statistical test(s) used AND whether they are one- or two-sided<br>*Only common tests should be described solely by name; describe more complex techniques in the Methods section.*
☐	☒	A description of all covariates tested
☐	☒	A description of any assumptions or corrections, such as tests of normality and adjustment for multiple comparisons
☐	☒	A full description of the statistical parameters including central tendency (e.g. means) or other basic estimates (e.g. regression coefficient) AND variation (e.g. standard deviation) or associated estimates of uncertainty (e.g. confidence intervals)
☐	☒	For null hypothesis testing, the test statistic (e.g. *F*, *t*, *r*) with confidence intervals, effect sizes, degrees of freedom and *P* value noted<br>*Give P values as exact values whenever suitable.*
☒	☐	For Bayesian analysis, information on the choice of priors and Markov chain Monte Carlo settings
☒	☐	For hierarchical and complex designs, identification of the appropriate level for tests and full reporting of outcomes
☐	☒	Estimates of effect sizes (e.g. Cohen's *d*, Pearson's *r*), indicating how they were calculated

Our web collection on statistics for biologists contains articles on many of the points above.

Software and code

Policy information about availability of computer code

Data collection

Whole-genome bisulfite sequencing (WGBS)
DNA amount was determined by Qubit. Sequencing libraries were assessed on a Bioanalyzer (Agilent Technologies) and quantified using the KAPA Library Quantification Kit (KAPA BIOSYSTEMS). The libraries were then sequenced on a HiSeq 2500 (Illumina) as 2× 100 bp or 2× 101 bp paired-end reads.

Target-captured bisulfite sequencing (Methyl-seq)
DNA amount was determined by Qubit. Sequencing libraries were assessed by Bioanalyzer and quantified using the KAPA Library Quantification Kit. The libraries were then sequenced on a HiSeq 2500 (Illumina) as 2× 100 bp or 2× 101 bp paired-end reads.

RNA sequencing
RNA quality was analyzed by Bioanalyzer. RNA-seq libraries were sequenced on a NextSeq500 (Illumina) as 75 bp single reads.

Data analysis

For WGBS and Methyl-seq analyses of 129/MSM genetic background cells and tissues, SNP data for MSM/Ms were obtained from the NIG Mouse Genome Database (MSMv4HQ, http://molossinus.lab.nig.ac.jp/msmdb/index.jsp), and the MSM/Ms mouse genome was reconstructed from mm10 using these SNPs. Information about indels was not used in this study. Bases with low-quality scores and the adapters in all sequenced reads were trimmed with cutadapt-1.14 (Martin, 2011). Trimmed reads were mapped independently to both the B6 (mm10) and MSM/Ms mouse genomes using the Bismark software-v0.18.2 (Krueger and Andrews, 2011) with bowtie2 (version 2.3.0) (Langmead and Salzberg, 2012). Reads uniquely mapped to the same chromosome and positions of both B6 and MSM/Ms genomes were used for further analyses. B6 (129)-derived and MSM/Ms-derived sequenced reads were determined based on MSM/Ms SNP data. The Y chromosome and mitochondrial genome were omitted from the MSM/Ms reference genome due to the lack of SNP data. SNPs in CpG sites were excluded, and the patterns of bisulfite conversion were taken into account in the determination of parental alleles. Methylated cytosines were extracted from reads using the Bismark methylation extractor with the following options: --ignore 10 --ignore_r2 10 --ignore_3prime 5 --ignore_3prime_r2 5. For Methyl-seq analysis of B6 genetic background tissues, trimmed reads were mapped to the mouse genome (mm10) using Bismark software-v0.18.2 with bowtie2 (version 2.3.0) and default settings, and uniquely

mapped reads were used for extraction of methylated cytosines using the Bismark methylation extractor with options --ignore 10 --ignore_3prime 5 (single-end reads) or --ignore 10 --ignore_r2 10 --ignore_3prime 5 --ignore_3prime_r2 5. Because probe sequences of the SureSelectXT were designed for the original top strand, only the original bottom (OB) data were used for this analysis. The R/Bioconductor package DSS (Wu et al., 2015) was used to identify differentially methylated loci and regions. For identification of epiblast-specific methylated loci (Fig. 1b), WGBS data obtained from GSE84236 were used with the DMLtest function to identify significantly differentially methylated regions (FDR <0.05, Epi > ICM). For identification of Dnmt3a and Dnmt3b target regions (Fig. 2a and Supplementary Fig. 3a), 10k tiled CpG methylation levels (CpG sites at ≥50× coverage) from Methyl-seq data were used with the DMLtest function to identify significantly differentially methylated tiles [FDR <0.01 and difference in methylation levels (WT - KO) >0.4]. For identification of Dnmt3a and Dnmt3b target genes (Supplementary Table 2-5), Methyl-seq data were used with the DMLtest and callDMR functions (delta = 0.2, p.threshold = 0.00001, minCG = 10) to identify significantly differentially methylated regions (DMRs). Genes included in the DMRs (WT > KO) were identified as target genes. The Integrative Genomics Viewer (IGV) (version 2.8.2)(Robinson et al., 2011) was used to visualize CpG methylated status (CpG sites at ≥5× coverage). Genomic locations for each genetic element were described previously (Illingworth et al., 2010). UCSC LiftOver (Rosenbloom et al., 2015) (http://genome.ucsc.edu/) was used to convert the coordinates of the mm9 assembly to those of the mm10 assembly.

For manuscripts utilizing custom algorithms or software that are central to the research but not yet described in published literature, software must be made available to editors/reviewers. We strongly encourage code deposition in a community repository (e.g. GitHub). See the Nature Research guidelines for submitting code & software for further information.

Data

Policy information about availability of data

All manuscripts must include a data availability statement. This statement should provide the following information, where applicable:

- Accession codes, unique identifiers, or web links for publicly available datasets
- A list of figures that have associated raw data
- A description of any restrictions on data availability

All data of WGBS, Methyl-seq, and RNA-seq used in this study were deposited in the Gene Expression Omnibus (GEO) under accession number GSE111172. Methyl-seq data of Dnmt3a WT tissue were deposited under accession number GSE111173. WGBS or Methyl-seq data of 2i/L ESCs, S/L ESCs and wild type 2i-MEFs were deposited under accession number GSE84165.

The publicly available datasets used in this study are:
GSE84236, GSE95747, GSE62298, GSE57413, GSE96529, GSE95747, GSE62298.

Field-specific reporting

Please select the one below that is the best fit for your research. If you are not sure, read the appropriate sections before making your selection.

☒ Life sciences ☐ Behavioural & social sciences ☐ Ecological, evolutionary & environmental sciences

For a reference copy of the document with all sections, see nature.com/documents/nr-reporting-summary-flat.pdf

Life sciences study design

All studies must disclose on these points even when the disclosure is negative.

Sample size	No statistical methods were used to predetermine sample size. Sample size was chosen based on standards in the field. Sample size is described in methods, figures or figure legends. qRT-PCR experiments were performed in biological triplicate. We have had enough number of sequencing reads for WGBS, RNA-seq and Methyl-seq. To obtain each Dnmt KO ESCs and MEFs, same parental female ESCs were used in this study. We have generated more than 3 independent Dnmt3a or Dnmt3b KO ESCs and MEFs lines and gotten genome wide sequencing data with duplicates.
Data exclusions	We don't have any exclusions in this study.
Replication	We have derived multiple WT, Dnmt3a KO, and Dnmt3b KO ESCs and MEFs. WGBS, Methyl-seq were performed using two independent materials. qRT-PCR experiments were performed in biological triplicate. We were able to confirm the reproducibility of our results.
Randomization	Randomization was not relevant. All cell lines or biological samples were analysed or treated in the same manner.
Blinding	Blinding was not possible due to the small number of samples.

Reporting for specific materials, systems and methods

We require information from authors about some types of materials, experimental systems and methods used in many studies. Here, indicate whether each material, system or method listed is relevant to your study. If you are not sure if a list item applies to your research, read the appropriate section before selecting a response.

Materials & experimental systems

n/a	Involved in the study	
☐	☒	Antibodies
☐	☒	Eukaryotic cell lines
☒	☐	Palaeontology
☐	☒	Animals and other organisms
☒	☐	Human research participants
☒	☐	Clinical data

Methods

n/a	Involved in the study	
☒	☐	ChIP-seq
☒	☐	Flow cytometry
☒	☐	MRI-based neuroimaging

Antibodies

Antibodies used

anti-Dnmt3a antibody (Novus Biological, NB120-13888, 64B1446; dilution, 1:500; Santa Cruz Biotechnology, sc-20703; dilution, 1:200)
anti-β-actin antibody (Santa Cruz Biotechnology, sc-47778; dilution, 1:1000)
ECL Anti-mouse IgG, HRP-linked whole antibody from sheep (GE Healthcare, NA931V; dilution, 1:5000)
ECL Anti-rabbit IgG, HRP-linked whole antibody from donkey (GE Healthcare, NA934V; dilution, 1:5000)

Validation

We have confirmed that we see a band of Dnmt3a in wild type cells and can't see it in Dnmt3a KO cells using the antibody. Also, those antibodies were used in our previous study (Yagi et al., Nature 2017). Here is links of antibodies; https://www.novusbio.com/products/dnmt3a-antibody-64b1446_nb120-13888, http://datasheets.scbt.com/sc-20703.pdf, https://www.scbt.com/scbt/product/beta-actin-antibody-c4.

Eukaryotic cell lines

Policy information about cell lines

Cell line source(s)

All wild type/ Dnmt KO mouse ES cell lines and MEFs (129X1/SvJ /MSM/Ms and 129X1/SvJ/C57BL/6) were established in our laboratory.

Authentication

We authenticated that each cell lines have correct genotypes using PCR, immunostaining and western blot.

Mycoplasma contamination

All samples used for animal experiments were tested for mycoplasma contamination. We confirmed that our cell lines are negative to mycoplasma contamination.

Commonly misidentified lines
(See ICLAC register)

No commonly misidentified lines were used in this study.

Animals and other organisms

Policy information about studies involving animals; ARRIVE guidelines recommended for reporting animal research

Laboratory animals

Genetic background of mouse cell lines were mixed between 129X1/SvJ and MSM/Ms or 129X1/SvJ and C57BL/6. We used female 129/SvJ (8-10 week old), male MSM/Ms and male C57BL/6 (8-10 week old). MSM/Ms were obtained from RIKEN Bio Resource Center and 129X1/SvJ and C57BL/6 were obtained from SLC. Pseudopregnant ICR and male and female ICR (8-10 week old) were obtained from SLC to make chimeric animals.B6;129S4-Dnmt3a<tm1Enl> mice (Dnmt3a hetero knockout) were obtained from RIKEN Bio Resource Center.

Wild animals

No wild animals were used in this study.

Field-collected samples

No field-collected samples were used in this study.

Ethics oversight

All experiments using animals were performed under the ethical guidelines of Kyoto University and University of Tokyo.

Note that full information on the approval of the study protocol must also be provided in the manuscript.
